# Microbial extracellular vesicles contribute to antimicrobial resistance

**DOI:** 10.1371/journal.ppat.1012143

**Published:** 2024-05-02

**Authors:** Bowei Jiang, Yi Lai, Wenhao Xiao, Tianyu Zhong, Fengping Liu, Junjie Gong, Junyun Huang

**Affiliations:** 1 The First School of Clinical Medicine, Gannan Medical University, Ganzhou, China; 2 Department of Laboratory Medicine, First Affiliated Hospital of Gannan Medical University, Ganzhou, China; La Trobe University College of Science Health and Engineering, AUSTRALIA

## Abstract

With the escalating global antimicrobial resistance crisis, there is an urgent need for innovative strategies against drug-resistant microbes. Accumulating evidence indicates microbial extracellular vesicles (EVs) contribute to antimicrobial resistance. Therefore, comprehensively elucidating the roles and mechanisms of microbial EVs in conferring resistance could provide new perspectives and avenues for novel antimicrobial approaches. In this review, we systematically examine current research on antimicrobial resistance involving bacterial, fungal, and parasitic EVs, delineating the mechanisms whereby microbial EVs promote resistance. Finally, we discuss the application of bacterial EVs in antimicrobial therapy.

## 1 Introduction

Antimicrobial resistance has emerged as a major public health issue worldwide in the 21st century, posing grave threats to human health and lives. Due to the overuse of antimicrobial medications, a growing number of pathogens have developed resistance to one or multiple antimicrobial agents [1,2]. With the rise of these multidrug-resistant microbes, many once-effective treatments can no longer address infectious diseases, resulting in extended hospitalizations, increased medical costs, and higher mortality rates [2]. According to data from the US Centers for Disease Control and Prevention (CDC), approximately 2 million people in the US are infected with antibiotic-resistant bacteria annually, directly causing at least 23,000 deaths each year [3]. Moreover, projections estimate antimicrobial resistance will cause a $100 trillion reduction in global GDP by 2050, presenting a substantial economic burden [4]. Therefore, combatting antimicrobial resistance is critical for safeguarding global public health.

Current research indicates that antimicrobial resistance is a complex phenomenon. Generally, pathogens can resist the effects of antimicrobial drugs through various mechanisms. These mechanisms include but are not limited to [5,6]: (1) degrading antimicrobial drugs through enzymes; (2) altering the structure of drug target to prevent the binding of antimicrobial drugs; (3) changing cell membrane permeability to prevent antimicrobial drugs from entering the cell; and (4) using efflux pumps to expel antimicrobial drugs from the cell, thereby reducing intracellular drug concentrations.

Notably, in recent research on antimicrobial resistance, extracellular vesicles (EVs) produced by microbes have garnered considerable scientific attention. Microbial EVs are nanoscale vesicles capable of transferring biomolecules like proteins, lipids, and nucleic acids from their parent cell, playing vital roles in inter-microbial communication, microbe–host interactions, and microbial adaptation to environmental stress [7,8]. Recent evidence indicates microbial EVs also significantly contribute to antimicrobial resistance. Consequently, this review summarizes the roles and mechanisms of bacterial, fungal, and parasitic EVs in promoting antimicrobial resistance, and discusses prospective applications of bacterial EVs in antimicrobial therapy.

## 2. Biogenesis and types of microbial EVs

**Fig 1:**
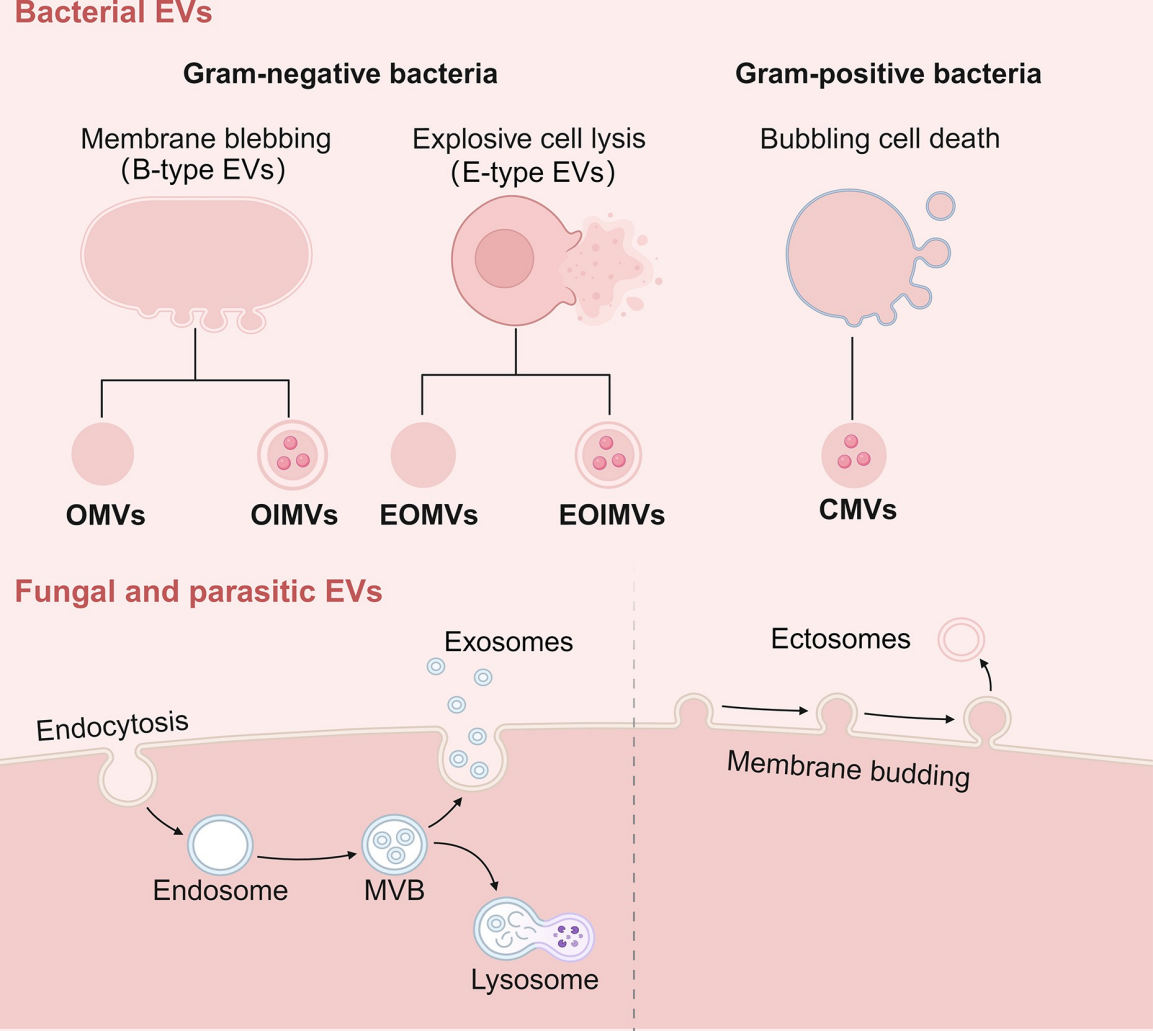
Types and biogenesis of bacterial, fungal, and parasitic EVs. Gram-negative bacteria can produce EVs through 2 mechanisms: membrane blebbing and explosive cell lysis. OMVs and OIMVs originate from membrane blebbing, while EOMVs and EOIMVs originate from explosive cell lysis. Gram-positive bacteria can produce CMVs through bubbling cell death. Additionally, fungi and parasites can produce EVs through 2 pathways: cell endocytosis and membrane budding. Exosomes are derived from cell endocytosis and ectosomes originate from membrane budding. EVs, extracellular vesicles; OMVs, outer membrane vesicles; OIMVs, outer-inner membrane vesicles; EOMVs, explosive outer membrane vesicles; EOIMVs, explosive outer-inner membrane vesicles; CMVs, cytoplasmic membrane vesicles. The figure was designed using Biorender.

Like other cells in the field of life, microbes can also spontaneously produce EVs. However, due to the diversity of microbial species, there are significant differences in the types and biogenesis of their EVs (Fig 1).

The current mainstream view is that the EVs produced by bacteria include: outer membrane vesicles (OMVs), outer-inner membrane vesicles (OIMVs), explosive outer membrane vesicles (EOMVs), explosive outer-inner membrane vesicles (EOIMVs), and cytoplasmic membrane vesicles (CMVs) [9]. Firstly, the formation of OMVs is due to the blebbling of the outer membrane in gram-negative bacteria. This type of EVs carries various outer membrane components and periplasmic contents, but is generally considered not to contain cytoplasmic components [10–12]. The formation of OIMVs is due to the blebbling of the inner and outer membranes in gram-negative bacteria, as a result of the weakening of the peptidoglycan layer by autolysins. This type of EVs has a double-membrane structure and is capable of carrying cytoplasmic components, such as plasmid DNA [13–15]. Notably, different from the blebbling mechanism of OMVs and OIMVs (B-type EVs), EOMVs and EOIMVs originate from the explosive cell lysis of gram-negative bacteria (E-type EVs). This occurs because phage-encoded endolysins degrade the peptidoglycan layer, leading to explosive lysis of cells, after which the membrane fragments spontaneously assemble into EOMVs and EOIMVs. These 2 types of EVs can randomly wrap cytoplasmic components, characterized by their ability to carry chromosomal DNA [16–18]. Additionally, in the case of CMVs, current researches suggest that endolysins, autolysins, or certain antibiotics that inhibit peptidoglycan synthesis create pores in the peptidoglycan cell wall of gram-positive bacteria, causing the cytoplasmic membrane to protrude outwardly to form CMVs. Although in this process, gram-positive bacteria do not undergo explosive lysis as gram-negative bacteria do, the loss of cytoplasmic membrane integrity still results in cell death. Hence, this mechanism is termed “bubbling cell death” [19–23].

It is noteworthy that compared to bacterial EVs, the EVs produced by fungi and parasites are more akin to the EVs of mammalian cells in types and biogenesis. The prevailing view holds that fungal and parasitic EVs can be classified into 2 categories: exosomes and ectosomes [24,25]. Exosomes range in size from 30 to 100 nm and their formation process involves: the plasma membrane invaginates to form endosomes, which then undergoes a second invagination to form multivesicular bodies (MVBs) containing intraluminal vesicles (ILVs). Finally, the MVBs fuse with the plasma membrane, releasing ILVs outside the cell as exosomes [26–28]. In contrast, ectosomes originate from the budding of the plasma membrane and range in size from 100 to 1,000 nm, including microvesicles, microparticles, and large vesicles [25,26].

## 3. Bacterial EVs contribute to antimicrobial resistance

**Fig 2:**
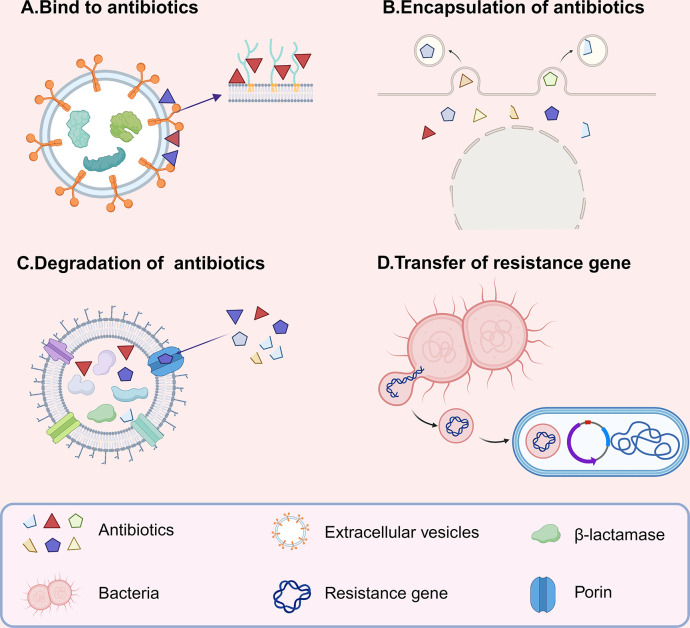
Bacterial EVs contribute to antibiotic resistance through multiple mechanisms. (A) EVs can act as decoys to bind to membrane-targeting antibiotics. (B) EVs can act as decoys to encapsulate antibiotics during formation. (C) EVs can degrade antibiotics through enzymes. (D) EVs can transfer resistance genes to recipient cells, mediating the spread of resistance. EVs, extracellular vesicles. The figure was designed using Biorender.

Research over the past few decades has confirmed that bacterial EVs are involved in various biological processes, including biofilm formation, delivery of virulence factors, stress responses, quorum signaling transduction, and nutrient acquisition [11,29]. Besides these functions, current research indicates that bacterial EVs can also contribute to antibiotic resistance through multiple mechanisms (Fig 2).

### 3.1. Bacterial EVs act as decoys to bind or encapsulate antibiotics

Firstly, the bacterial cell membrane is primarily composed of a phospholipid bilayer. This membrane structure not only provides a protective barrier for bacteria but also control the influx and efflux of substances [30,31]. However, certain antibiotics that target the membrane, such as daptomycin and polymyxin B, can bind to phospholipids or lipopolysaccharides (LPS) on the bacterial cell membrane, respectively, thereby disrupting membrane integrity and leading to bacterial death [32,33]. Recent studies have revealed that bacterial EVs can act as “decoys” to bind to these membrane-targeting antibiotics. For example, OMVs secreted by *Escherichia coli* can bind to polymyxin B to protect bacteria from polymyxin B [34]. CMVs produced by *Staphylococcus aureus* can bind to daptomycin, effectively preserving bacterial survival [22]. Likewise, OMVs from *Pseudomonas syringae* Lz4W and *E*. *coli* MG1655 can bind to colistin and the antimicrobial peptide melittin, attenuating their toxic effects. However, it is important to note that the OMVs from these 2 types of bacteria cannot protect bacteria from non-β-lactam antibiotics that act on other targets, such as ciprofloxacin, streptomycin, and trimethoprim [35,36].

From the above examples, it is evident that both OMVs and CMVs can act as decoys to bind to membrane-targeting antibiotics, thereby providing protection. This may be attributed to the membrane origin of bacterial EVs, which provides binding targets for interaction with membrane-targeting antibiotics, such as LPS and phospholipids. However, for antibiotics that act on other targets, EVs do not have corresponding binding sites to interact with. Therefore, it is evident that the ability of bacterial EVs to bind to antibiotics is mainly related to the types of antibiotics, rather than the types of EVs.

Additionally, it is noteworthy that some recent studies have observed an interesting phenomenon: when bacteria are exposed to antibiotics, they will release EVs carrying antibiotics. For instance, Kadurugamuwa and colleagues [37,38] discovered that *Pseudomonas aeruginosa* and *Shigella flexneri*, after exposure to gentamicin, produce more EVs, and gentamicin was detected in these EVs. Furthermore, the authors confirmed that gentamicin was located in the lumen of EVs by immunogold labeling. Similarly, Huang and colleagues [39] demonstrated that *Acinetobacter baumannii*, under stress from subinhibitory concentrations of levofloxacin, also produce EVs carrying levofloxacin. The authors used EDTA to disrupt the membrane of the EVs and analyzed the form in which levofloxacin was encapsulated. They found that compared to the untreated group, significant amounts of levofloxacin were only detectable in the EVs after EDTA treatment. This suggests that levofloxacin was encapsulated inside the EVs. Moreover, this phenomenon has also been observed in mycoplasmas. Medvedeva and colleagues [40] showed that ciprofloxacin treatment can induce *Acholeplasma laidlawii* to produce EVs carrying ciprofloxacin.

It is important to note that although the aforementioned studies confirm that bacterial EVs can encapsulate antibiotics, how bacterial EVs perform this encapsulation remains a puzzle. In the study by Huang and colleagues mentioned above, the authors found that subinhibitory concentrations of levofloxacin can induce high expression of efflux pump proteins adeB, adeA, and acrB in *A*. *baumannii*. However, the expression of outer membrane protein adeC and porin proteins in the efflux pump system was significantly reduced. Therefore, the authors suggest that bacteria expel antibiotics out of the cell in the form of OMVs through the high expression of efflux pumps [39]. Additionally, considering the biogenesis mechanism of bacterial EVs, the encapsulation of antibiotics by EVs is likely a natural process during EV formation. For example, antibiotics like gentamicin, which interact with bacterial LPS, could lead to membrane bending [10]. This may result in the encapsulation of antibiotics in OMVs. Moreover, antibiotics like ciprofloxacin that damage DNA can induce a bacterial SOS response, activating endolysin expression and resulting in explosive cell lysis [41]. As a result, EOIMVs and EOMVs may encapsulate antibiotics during their assembly process.

### 3.2. Bacterial EVs degrade antibiotics through antibiotic-degrading enzymes

It is noteworthy that current research indicates that bacterial EVs can transport β-lactamases outside the cell [42–44]. This occurs because when bacteria producing β-lactamases are under antibiotic stress, they can employ the Sec system to transport β-lactamases to the periplasmic space. Subsequently, the β-lactamases are encapsulated into EVs through favorable electrostatic protein–membrane interactions and then transported outside the cell [45,46]. Many examples have shown that these β-lactamases-carrying EVs can degrade surrounding antibiotics to protect bacteria. For instance, EVs from *Haemophilus influenzae* and *Moraxella catarrhalis* harbor active β-lactamases and protect *Streptococcus pneumoniae*, *H*. *influenzae*, and *Group A streptococci* by hydrolyzing amoxicillin [47–49]. EVs from *Bacteroides* spp. contain cephalosporinases and can protect intestinal bacteria including *Salmonella* and *Bifidobacteria* by degrading cefotaxime [50]. Moreover, EVs from methicillin-resistant *S*. *aureus* (MRSA) also utilize β-lactamases to degrade β-lactam antibiotics, thereby safeguarding *E*. *coli* [51].

It is important to note that this degradation process requires the involvement of porins on the surface of bacterial EVs. Porins are proteins that form channels on the bacterial outer membrane, allowing small molecules to enter the bacteria [52]. Hence, bacterial EVs absorb surrounding antibiotics through these porins and then internal β-lactamases hydrolyze antibiotics. In the absence of porins, EVs exhibit a significantly diminished antibiotic degradation capability [53]. Additionally, research has shown that bacterial EVs can promote bacterial porin mutations by decreasing antibiotic concentrations. For example, Zhang and colleagues [54] demonstrated that when carbapenem-sensitive *P*. *aeruginosa* was co-cultured with low concentrations of carbapenemase-containing EVs and imipenem, mutations were induced in the *P*. *aeruginosa* outer membrane porin OprD. This eventually resulted in the bacterium developing resistance to carbapenem antibiotics.

### 3.3. Bacterial EVs transfer resistance genes to recipient cell

The above researches show that EVs can help bacteria resist antibiotic stress through various mechanisms. More importantly, EVs can also mediate the spread of resistance by transferring resistance genes. However, it should be noted that current researches propose that classic OMVs typically do not carry DNA and only OIMVs, EOMVs, EOIMVs, and CMVs have the chance to carry DNA [9]. This is because the biogenesis mechanism of OMVs only involves bacterial outer membrane, thus they cannot carry cytoplasmic contents [9].

There are already many examples showing that bacterial EVs can transfer resistance genes both interspecies and intraspecies. For example, EVs from carbapenem-resistant *klebsiella pneumoniae* can transfer *blaKPC-2* and *blaNDM-1* resistance genes to susceptible *K*. *pneumoniae*, including highly virulent *K*. *pneumoniae*, thereby conferring carbapenem resistance [55,56]. Similarly, EVs from carbapenem-resistant *A*. *baumannii* can transfer plasmids carrying *blaOXA-24* and *blaNDM-1* resistance genes to susceptible *A*. *baumannii* and *E*. *coli*, conferring carbapenem resistance [57,58]. In addition, EVs from avian pathogenic *E*. *coli* and *E*. *coli* O104:H4 can transfer plasmids carrying *blaCTX-M-15* resistance genes to other Enterobacteriaceae, enabling them to express extended-spectrum β-lactamases [59,60]. Through the above examples, it is evident that bacterial EVs can transfer resistance genes among different types of bacteria. However, a study by Renelli and colleagues pointed out that although the EVs of *P*. *aeruginosa* PAO1 carried the plasmid pAK1900, they did not successfully transfer the plasmid to the recipient strains PAO1 and *E*. *coli* DH5α [18]. The authors speculated that this might be due to the inability of the plasmid to cross the inner membrane. Regarding this issue, Fulsundar and colleagues [61] indicates that in *Acinetobacter baylyi*, EVs containing DNA lyse upon contact with the bacterial outer membrane, after which the DNA is transported into the cytoplasm by type IV pili. This suggests that the uptake of EV-associated DNA may depend on the competence machineries of the recipients.

Moreover, it should be noted that not every EV-mediated transfer of resistance genes can stably induce a resistant phenotype. For example, Xu and colleagues [62] co-cultured EVs from *Avibacterium paragallinarum* P4chr1, which carried resistance genes, with the susceptible strain *A*. *paragallinarum* Modesto. Although they successfully screened for transformed colonies on antibiotic-containing plates, susceptibility tests revealed that the transferred resistance genes were not stable in the recipient cells, as the bacteria lost their resistance after a second passage. The authors speculated that this might be due to the absence of homologous recombination of the resistance genes in the recipient cells. However, this result differs from the findings of Chen and colleagues, who showed that EVs from carbapenem-resistant *K*. *pneumoniae* can induce stable resistance in carbapenem-susceptible *K*. *pneumoniae*, and this resistance was maintained beyond the third generation [55].

It is worth noting that currently some studies have also pointed out that certain factors can influence EV-mediated DNA transfer. For example, Tran and colleagues [63] assessed the impact of plasmid characteristics on EV-mediated DNA transfer. It was found that the higher the plasmid copy number, the higher the DNA loading in EVs, and the shorter the gene transfer time. Furthermore, Fulsundar and colleagues [61] showed that EVs from *A*. *baylyi* isolated in the presence of gentamicin transferred plasmid DNA to recipient cells 10 times more efficiently than EVs isolated without antibiotic exposure. The authors believe this may be due to gentamicin altering the surface potential of EVs, leading to easier interaction between EVs and recipient cells. In addition, Johnston and colleagues [64] showed that EVs produced by *P*. *aeruginosa* under biofilm conditions are smaller but contain more plasmid DNA compared to those produced under planktonic conditions. Biofilm-derived *P*. *aeruginosa* EVs could more efficiently transfer plasmids carrying resistance genes to recipient cells.

## 4. Fungal EVs contribute to antimicrobial resistance

In addition to bacterial resistance, fungal resistance also presents a significant challenge to global health, posing a severe threat to the treatment outcomes and prognosis of fungal infections. Moreover, the range of antifungal drug options is limited, with only a few classes of antifungal drugs available, underscoring the urgent need for effective antifungal management strategies to control fungal resistance. Currently, there is mounting evidence suggesting that fungal EVs play a pivotal role in fungal drug resistance.

First, studies have shown that fungal biofilm-derived EVs contribute to the resistance of biofilms against antifungal drugs. For example, Kulig and colleagues [65] found that in the presence of the antifungal drug caspofungin, the addition of *Candida tropicalis* biofilm EVs at the initial stage of the formation of *C*. *tropicalis* biofilm can increase the thickness of the biofilm as well as the metabolic activity and viability of biofilm cells, which means that biofilm EVs are involved in resistance to antifungal drugs. Additionally, in a series of studies on *Candida albicans* by Zarnowski and colleagues, it was found that biofilm EVs are enriched with proteins, lipids, and polysaccharides similar to the biofilm matrix, which can protect the biofilm cells by isolating antimicrobial drugs. Using endosomal sorting complexes required for transport (ESCRT) mutants, the authors demonstrated the critical role of biofilm EVs in matrix production and function. The ESCRT mutants showed a reduction in biofilm EV production, decreased matrix polysaccharides, and increased sensitivity to antifungal drugs. Adding wild-type biofilm EVs to ESCRT mutant biofilms could restore matrix accumulation and drug resistance [66–68]. Similarly, Zhao and colleagues [69] reported that inhibition of fungal vesicle-mediated trafficking can greatly weaken the assembly of the biofilm matrix. This study found that an antifungal drug turbinmicin can inhibit the production of various fungal biofilm EVs, which greatly weakens the biofilm matrix, resulting in drug-resistant biofilm communities easy to be killed by drugs, and the addition of exogenous biofilm EVs could restore the resistance of biofilms against drugs. In conclusion, fungal EVs are critical to the assembly of biofilm matrix, which promotes biofilm resistance mainly by delivering biofilm matrix materials.

Secondly, fungal EVs are also involved in cell wall repair and remodeling, as fungal EVs carry cell wall-associated proteins. A study has shown that when *Candida auris* is treated with caspofungin, the EV production significantly increases. At the same time, this treatment changed the protein composition within EVs, including proteins associated with the cell wall [70]. In addition, Zhao and colleagues [71] found that *saccharomyces cerevisiae* with defects in cell wall synthesis release a large amount of EVs rich in cell wall-synthesizing enzymes, such as glucan synthase subunits Fks1 and chitin synthase Chs3. These EVs can be absorbed by other yeast cells, thereby protecting them from the toxic effects of caspofungin. Similarly, Martinez-Lopez and colleagues [72] showed that EVs produced by *C*. *albicans* yeast-like cells are rich in cell wall proteins, and these EVs can protect cell wall mutant strains from the effects of the cell wall inhibitor calcofluor white. Interestingly, in a study by Chan and colleagues, it was found that *C*. *auris* EVs can dose-dependently increase the resistance of *C*. *auris* to amphotericin B, but have no effect on the sensitivity of *C*. *albicans* to amphotericin B. The authors speculated that *C*. *auris* EVs may regulate the resistance to amphotericin B by supplementing membrane materials or providing cell wall repair enzymes. However, this species-specific mechanism warrants further investigation [73].

Moreover, fluconazole (FLC), a widely used antifungal drug, is facing escalating resistance [74–76]. Typically, fungi achieve resistance to FLC by altering the drug’s target or increasing the expression of drug efflux pumps [77,78]. However, a recent study by Rizzo and colleagues revealed a new finding. They observed that *Cryptococcus neoformans* could adapt to FLC pressure by regulating EV production rather than by changing the drug target or drug efflux pump expression. In this study, the authors identified 4 transcription factors (*HAP2*, *GAT5*, *LIV4*, and *BZP2*) that significantly affect EV production, through a transcription factor mutant library. These transcription factor mutants showed altered sensitivity to FLC along with reduced EV production. Additionally, the authors analyzed the changes in EV production when FLC resistance was acquired or lost in clinical isolates, finding a co-regulation of FLC resistance and EV production. Therefore, the authors propose a new perspective that *C*. *neoformans* can adapt to FLC pressure by regulating EV production [79].

## 5. Parasitic EVs contribute to antimicrobial resistance

In recent years, researchers have increasingly recognized the importance of EVs from pathogenic parasites, which play a significant role in the pathogenicity, transmission, and immune modulation of these parasites [80]. During parasitic infections, EVs released by parasites can directly induce signaling pathways in host cells, thereby affecting the host’s immune response [81,82]. Notably, emerging researches have also begun to reveal the role of parasitic EVs in drug resistance.

Initially, Regev-Rudzki and colleagues [83] showed that *Plasmodium falciparum* can transfer drug resistance genes to other *P*. *falciparum* through EVs, leading to the spread of drug resistance. In this study, the authors engineered 2 types of *P*. *falciparum* with different drug-resistant plasmids, resistant to the antimalarial drugs blasticidin (Bs) and WR99210 (WR), respectively. Through co-cultivation experiments, the authors found that the *P*. *falciparum* infecting red blood cells could not survive alone in the culture media containing WR and Bs. Only when the 2 types of *P*. *falciparum* were mixed in culture did they survive, indicating the transfer of resistance genes between them. Subsequently, the authors discovered the presence of approximately 70 nm EVs in the mixed culture fluid, and their quantity increased with the escalation of drug pressure. Finally, the authors used a series of sophisticated experimental designs to demonstrate that the transfer of resistance genes does not require physical contact but is dependent on the secretion and uptake of EVs.

Subsequently, Douanne and colleagues [84] also reported the mechanism of gene exchange through EVs in *Leishmania*. The team confirmed the enrichment of resistance genes in EVs from drug-resistant *Leishmania* by DNA sequencing and PCR, and EVs could effectively transfer resistance genes to sensitive *Leishmania*, leading to increased drug resistance. Notably, this transfer occurred not only between the same species of *Leishmania* but also between different species. In addition, they found that EVs from drug-resistant *Leishmania* could reduce oxidative stress in recipient *Leishmania*, promoting their growth and adaptability.

From the above studies, it is evident that EVs can transfer genes between parasites. However, it should be noted that EVs can also transfer proteins between parasites. For instance, Szempruch and colleagues [85] found that EVs secreted by *Trypanosoma brucei rhodesiense* are rich in serum resistance-associated protein (SRA), which can counteract trypanosome lytic factors (TLF). Through co-cultivation experiments, this study confirmed that *T*. *b*. *rhodesiense* EVs can transfer SRA to TLF-sensitive trypanosomes, conferring TLF resistance. Additionally, Douanne and colleagues [86] compared EVs from drug-resistant infant *Leishmania* strains with those from wild-type strains, noting that the drug resistance mechanism affects the morphology, size, and protein content of EVs. Their proteomics analysis revealed that EVs from different drug-resistant strains are enriched with proteins related to drug resistance. However, further exploration is needed to understand the other functions of these EVs carrying drug-resistant proteins.

## 6. The prospect of bacterial EVs in antimicrobial therapy

**Fig 3:**
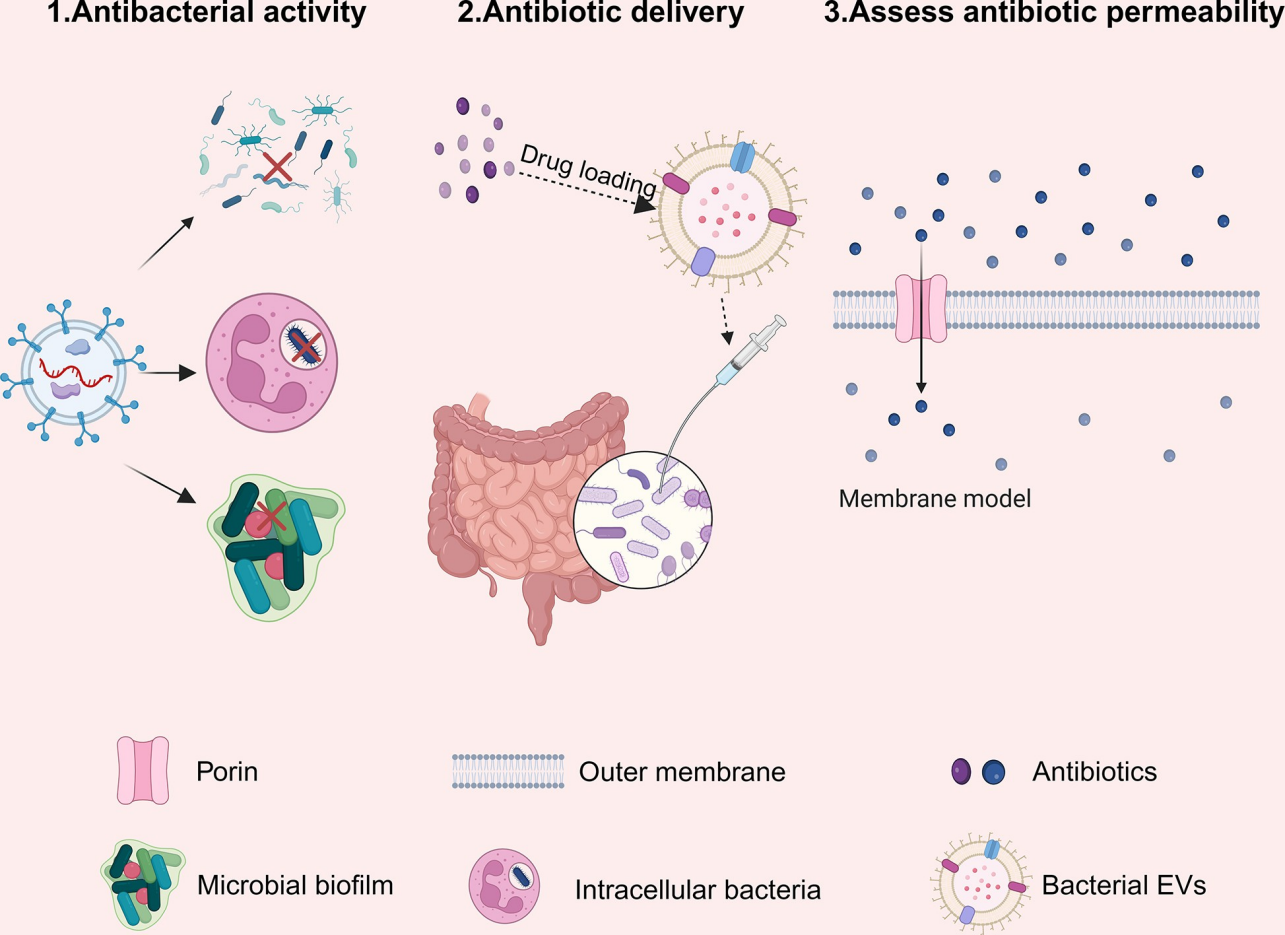
Bacterial EVs contribute to antimicrobial therapy. (1) Bacterial EVs may possess natural antibacterial activity to directly kill pathogens. (2) Bacterial EVs can be loaded with antibiotics, enhancing the targeting, affinity and stability of antibiotics. (3) Bacterial EVs can mimic the bacterial outer membrane and be used to assess the permeability of antibiotics. EVs, extracellular vesicles. The figure was designed using Biorender.

Amidst the rampant spread of antibiotic-resistant bacteria, the search for novel antimicrobial materials has gradually become a research focus. As a natural biomaterial, EVs have inevitably caught the attention of researchers. Recent studies have shown that bacterial EVs have potential application value in antimicrobial therapy (Fig 3).

### 6.1. Bacterial EVs has natural antimicrobial activity

When bacteria compete with other microorganisms for survival conditions, they can secrete antibacterial substances to inhibit their competitors, which is a survival strategy for some bacteria [87]. EVs, as a communication tool for bacteria, are naturally considered to carry antibacterial substances. Initially, Kadurugamuwa and colleagues [37] found that *P*. *aeruginosa* PAO1 EVs showed bactericidal activity against other bacteria, including both gram-negative and gram-positive bacteria. This bactericidal activity of EVs might rely on the synergistic action of different hydrolases [88]. Subsequently, more and more studies have shown that EVs secreted by other bacteria also have strong natural bactericidal activity. For example, EVs from the *Burkholderia thailandensis* contain peptidoglycan hydrolases, 4-hydroxy-3-methyl-2-(2-non-enyl)-quinoline (HMNQ), and rhamnolipid, which can inhibit the growth and biofilm formation of MRSA [89]. In contrast, EVs from *Myxococcus xanthus* contain various proteases, alkaline phosphatases, and secondary metabolites, which can kill *E*. *coli* and *P*. *aeruginosa* [90].

Moreover, it is worth noting that in clinical treatment, how to effectively treat intracellular bacterial infections remains a significant challenge. For instance, at the same dose, planktonic MRSA can be killed by antibiotics, while intracellular bacteria can continue to survive and replicate [91]. In response to this issue, Goes and colleagues [92] reported that *myxobacteria* EVs could be easily phagocytosed by lung epithelial cells and macrophages, delivering the antibacterial substance cystobactamid into infected cells to inhibit intracellular *S*. *aureus*. Moreover, these *myxobacteria* EVs are non-toxic to human cells and do not cause excessive inflammatory responses.

In addition to antibacterial activity, there are also reports on the antifungal activity of bacterial EVs. For instance, Meers and colleagues [93] reported that *Lysobacter enzymogenes* C3 EVs contain various small molecule compounds with antifungal activity, such as dihydromaltophilin and alteramide B and can deliver antifungal compounds into *Saccharomyces cerevisiae* to inhibit their growth. Moreover, Huang and colleagues [94] discovered that *L*.*enzymogenes* OH11 can produce antifungal compounds HSAF and lytic polysaccharide monooxygenase. These 2 substances are encapsulated in EVs, acting on the fungal cell wall through EVs, thereby inhibiting the growth of fungi. It is evident from these studies that the potency of bacterial EVs in combatting pathogens stems from their enzymatic activity. Notably, these EVs offer a robust cell fusion capability—an attribute surpassing most conventional antibiotics. The potential of natural bacterial EVs in antibacterial therapies should be a focal point in future treatments.

### 6.2. Bacterial EVs are useful for antibiotic delivery

Additionally, bacterial EVs have the capability to encapsulate various types of antibiotics due to their hydrophobic membranes and hydrophilic cavities [95]. More importantly, bacterial EVs exhibit excellent stability, targeting capability, and biocompatibility [96,97]. Firstly, Huang and colleagues [39] utilized the mechanism of antibiotic encapsulation mediated by EVs from *A*. *baumannii* to prepare EVs loaded with quinolone antibiotics (levofloxacin, ciprofloxacin, and norfloxacin). Then, the bactericidal ability of these antibiotic-loaded EVs were tested in vitro and in vivo, confirming that they can effectively kill pathogens. Moreover, in a mouse intestinal infection model, a single low-dose oral administration could continuously release the drug at the infection site for 36 h, significantly reducing the number of bacteria in the infected mice’s intestines and feces. Additionally, these antibiotic-loaded EVs did not cause cytotoxicity or immune responses to intestinal epithelial cells, macrophages, and symbiotic bacteria, indicating that bacterial EVs possess excellent targeting specificity.

Regarding the targeting phenomenon of antibiotic-loaded EVs, Tashiro and colleagues [98] found that *Buttiauxella agrestis* EVs loaded with gentamicin could specifically bind to *B*. *agrestis* cells to kill them. To explain this, the group employed the Derjaguin–Landau–Verwey–Overbeek (DLVO) theory, which is used to calculate the total of van der Waals attraction and electrostatic repulsion between EVs and cells. They calculated the interaction energy between *B*. *agrestis* EVs and cells, finding that the interaction energy between *B*. *agrestis* EVs and cells of the same bacterial species was significantly lower than with other bacterial species. This might be the reason for the specificity of antibiotic-loaded EVs targeting bacteria of the same species. In conclusion, it is evident that bacterial EVs offer targeting, stability, and high-affinity in antibiotic delivery. Using bacterial EVs to load antibiotics can not only effectively deliver the antibiotic to bacteria but also develop targeted treatments for various bacterial infections.

### 6.3. Bacterial EVs as a method to assess antibiotic permeability

Moreover, for some antibiotics that act on intracellular targets, the bacterial cell membrane is a major barrier to their entry into the cell [99,100]. Since bacterial EVs can effectively mimic the physiological characteristics of the bacterial outer membrane, using bacterial EVs to assess antibiotic permeability is an effective method. Initially, Richter and colleagues [101] proposed an antibiotic permeation experiment based on bacterial OMVs. The authors isolated OMVs from *E*. *coli* and coated them onto polycarbonate filter supports, creating a membrane model that simulates the outer membrane of cells. They then conducted permeation experiments on this membrane model with 9 different types of antibiotics and correlated the obtained permeability coefficients with previously reported data on bacterial accumulation. It was found that the OMV-based membrane model could effectively differentiate between high and low permeability antibiotics and had a good correlation with the data on bacterial accumulation.

In addition, Ferreira and colleagues [102] also developed an experimental method using OMVs to measure the antibiotic permeability. Specifically, the authors placed OMVs in a high-concentration solution containing antibiotics and used dynamic light scattering (DLS) technology to measure the swelling rate of OMVs for 14 small molecule drugs. They then evaluated the relative permeability of the drugs through the OMVs based on the degree of swelling rate change. The authors found that the OMVs swelling rate correlated highly with the drug permeation coefficient predicted by molecular dynamics simulation (R = 0.97). These results confirm the reliability of measuring drug permeability using OMVs. Overall, bacterial EVs offer a simple, direct, and reproducible measurement method that helps in screening and optimizing drugs with high permeability.

## 7. Conclusions

In summary, microbial EVs play a significant role in antimicrobial resistance, which brings additional challenges to the treatment of pathogenic microbial infections. EVs from bacteria, fungi, and parasites can contribute to antimicrobial resistance directly or indirectly. Firstly, under the stress of antibiotics, bacterial EVs can act as decoys to bind and encapsulate antibiotics or degrade antibiotics through enzymes. In addition, both bacteria and parasites can transfer resistance genes to recipient cells through EVs, thereby promoting the spread of resistance. In fungi, fungal EVs primarily promote resistance by participating in the biogenesis of the biofilm matrix or in the repair and remodeling of the cell wall. In conclusion, these microbial EVs can contribute to antimicrobial resistance through various mechanisms, providing a new perspective for the formation and spread of microbial drug resistance. Looking ahead, bacterial EVs, as a natural biomolecule, also show great potential in antimicrobial therapy. For instance, leveraging their advantages in antibiotic delivery could open up new antimicrobial treatment strategies. However, there are still technical limitations in current applications, such as the difficulty of large-scale preparation of EVs and surface modification. Therefore, further breakthroughs are needed in subsequent research.

## References

[1] Ventola CL. The antibiotic resistance crisis: part 1: causes and threats. P T. 2015;40(4):277–83. Epub 2015/04/11. ; PubMed Central PMCID: PMC4378521. 25859123 PMC4378521

[2] Medina E, Pieper DH. Tackling Threats and Future Problems of Multidrug-Resistant Bacteria. Curr Top Microbiol Immunol. 2016;398:3–33. Epub 2016/07/14. doi: 10.1007/82_2016_492 . 27406189

[3] Brinkac L, Voorhies A, Gomez A, Nelson KE. The Threat of Antimicrobial Resistance on the Human Microbiome. Microb Ecol. 2017;74(4):1001–8. Epub 2017/05/12. doi: 10.1007/s00248-017-0985-z ; PubMed Central PMCID: PMC5654679. 28492988 PMC5654679

[4] Liu X, Xiao J, Wang S, Zhou J, Qin J, Jia Z, et al. Research Progress on Bacterial Membrane Vesicles and Antibiotic Resistance. Int J Mol Sci. 2022;23(19). Epub 2022/10/15. doi: 10.3390/ijms231911553 ; PubMed Central PMCID: PMC9569563. 36232856 PMC9569563

[5] Darby EM, Trampari E, Siasat P, Gaya MS, Alav I, Webber MA, et al. Molecular mechanisms of antibiotic resistance revisited. Nat Rev Microbiol. 2023;21(5):280–95. Epub 2022/11/22. doi: 10.1038/s41579-022-00820-y . 36411397

[6] Fisher MC, Alastruey-Izquierdo A, Berman J, Bicanic T, Bignell EM, Bowyer P, et al. Tackling the emerging threat of antifungal resistance to human health. Nat Rev Microbiol. 2022;20(9):557–71. Epub 2022/03/31. doi: 10.1038/s41579-022-00720-1 ; PubMed Central PMCID: PMC8962932. 35352028 PMC8962932

[7] Schertzer JW, Whiteley M. Bacterial outer membrane vesicles in trafficking, communication and the host-pathogen interaction. J Mol Microbiol Biotechnol. 2013;23(1–2):118–30. Epub 2013/04/26. doi: 10.1159/000346770 . 23615200

[8] Tsatsaronis JA, Franch-Arroyo S, Resch U, Charpentier E. Extracellular Vesicle RNA: A Universal Mediator of Microbial Communication? Trends Microbiol. 2018;26(5):401–10. Epub 2018/03/20. doi: 10.1016/j.tim.2018.02.009 . 29548832

[9] Toyofuku M, Schild S, Kaparakis-Liaskos M, Eberl L. Composition and functions of bacterial membrane vesicles. Nat Rev Microbiol. 2023;21(7):415–30. Epub 2023/03/19. doi: 10.1038/s41579-023-00875-5 . 36932221

[10] Kadurugamuwa JL, Clarke AJ, Beveridge TJ. Surface action of gentamicin on Pseudomonas aeruginosa. J Bacteriol. 1993;175(18):5798–805. Epub 1993/09/01. doi: 10.1128/jb.175.18.5798-5805.1993 ; PubMed Central PMCID: PMC206658. 8376327 PMC206658

[11] Kulp A, Kuehn MJ. Biological functions and biogenesis of secreted bacterial outer membrane vesicles. Annu Rev Microbiol. 2010;64:163–84. Epub 2010/09/10. doi: 10.1146/annurev.micro.091208.073413 ; PubMed Central PMCID: PMC3525469. 20825345 PMC3525469

[12] Schwechheimer C, Kuehn MJ. Outer-membrane vesicles from Gram-negative bacteria: biogenesis and functions. Nat Rev Microbiol. 2015;13(10):605–19. Epub 2015/09/17. doi: 10.1038/nrmicro3525 ; PubMed Central PMCID: PMC5308417. 26373371 PMC5308417

[13] Perez-Cruz C, Carrion O, Delgado L, Martinez G, Lopez-Iglesias C, Mercade E. New type of outer membrane vesicle produced by the Gram-negative bacterium Shewanella vesiculosa M7T: implications for DNA content. Appl Environ Microbiol. 2013;79(6):1874–81. Epub 2013/01/15. doi: 10.1128/AEM.03657-12 ; PubMed Central PMCID: PMC3592255. 23315742 PMC3592255

[14] Perez-Cruz C, Delgado L, Lopez-Iglesias C, Mercade E. Outer-inner membrane vesicles naturally secreted by gram-negative pathogenic bacteria. PLoS ONE. 2015;10(1):e0116896. Epub 2015/01/13. doi: 10.1371/journal.pone.0116896 ; PubMed Central PMCID: PMC4291224. 25581302 PMC4291224

[15] Aktar S, Okamoto Y, Ueno S, Tahara YO, Imaizumi M, Shintani M, et al. Incorporation of Plasmid DNA Into Bacterial Membrane Vesicles by Peptidoglycan Defects in Escherichia coli. Front Microbiol. 2021;12:747606. Epub 2021/12/17. doi: 10.3389/fmicb.2021.747606 ; PubMed Central PMCID: PMC8667616. 34912309 PMC8667616

[16] Baeza N, Delgado L, Comas J, Mercade E. Phage-Mediated Explosive Cell Lysis Induces the Formation of a Different Type of O-IMV in Shewanella vesiculosa M7(T). Front Microbiol. 2021;12:713669. Epub 2021/10/26. doi: 10.3389/fmicb.2021.713669 ; PubMed Central PMCID: PMC8529241. 34690958 PMC8529241

[17] Turnbull L, Toyofuku M, Hynen AL, Kurosawa M, Pessi G, Petty NK, et al. Explosive cell lysis as a mechanism for the biogenesis of bacterial membrane vesicles and biofilms. Nat Commun. 2016;7:11220. Epub 2016/04/15. doi: 10.1038/ncomms11220 ; PubMed Central PMCID: PMC4834629. 27075392 PMC4834629

[18] Renelli M, Matias V, Lo RY, Beveridge TJ. DNA-containing membrane vesicles of Pseudomonas aeruginosa PAO1 and their genetic transformation potential. Microbiology (Reading). 2004;150(Pt 7):2161–9. Epub 2004/07/17. doi: 10.1099/mic.0.26841-0 . 15256559

[19] Toyofuku M, Carcamo-Oyarce G, Yamamoto T, Eisenstein F, Hsiao CC, Kurosawa M, et al. Prophage-triggered membrane vesicle formation through peptidoglycan damage in Bacillus subtilis. Nat Commun. 2017;8(1):481. Epub 2017/09/09. doi: 10.1038/s41467-017-00492-w ; PubMed Central PMCID: PMC5589764. 28883390 PMC5589764

[20] Liu Y, Tempelaars MH, Boeren S, Alexeeva S, Smid EJ, Abee T. Extracellular vesicle formation in Lactococcus lactis is stimulated by prophage-encoded holin-lysin system. Microb Biotechnol. 2022;15(4):1281–95. Epub 2022/03/02. doi: 10.1111/1751-7915.13972 ; PubMed Central PMCID: PMC8966010 35229476 PMC8966010

[21] Mitchell GJ, Wiesenfeld K, Nelson DC, Weitz JS. Critical cell wall hole size for lysis in Gram-positive bacteria. J R Soc Interface. 2013;10(80):20120892. Epub 2013/01/11. doi: 10.1098/rsif.2012.0892 ; PubMed Central PMCID: PMC3565739. 23303219 PMC3565739

[22] Andreoni F, Toyofuku M, Menzi C, Kalawong R, Mairpady Shambat S, Francois P, et al. Antibiotics Stimulate Formation of Vesicles in Staphylococcus aureus in both Phage-Dependent and -Independent Fashions and via Different Routes. Antimicrob Agents Chemother. 2019;63(2). Epub 2018/12/05. doi: 10.1128/AAC.01439-18 ; PubMed Central PMCID: PMC6355553. 30509943 PMC6355553

[23] Abe K, Toyofuku M, Nomura N, Obana N. Autolysis-mediated membrane vesicle formation in Bacillus subtilis. Environ Microbiol. 2021;23(5):2632–47. Epub 2021/04/06. doi: 10.1111/1462-2920.15502 . 33817925

[24] Nenciarini S, Cavalieri D. Immunomodulatory Potential of Fungal Extracellular Vesicles: Insights for Therapeutic Applications. Biomolecules. 2023;13(10). Epub 2023/10/28. doi: 10.3390/biom13101487 ; PubMed Central PMCID: PMC10605264. 37892168 PMC10605264

[25] Rodrigues ML, Godinho RM, Zamith-Miranda D, Nimrichter L. Traveling into Outer Space: Unanswered Questions about Fungal Extracellular Vesicles. PLoS Pathog. 2015;11(12):e1005240. Epub 2015/12/04. doi: 10.1371/journal.ppat.1005240 ; PubMed Central PMCID: PMC4669077. 26633018 PMC4669077

[26] Szempruch AJ, Dennison L, Kieft R, Harrington JM, Hajduk SL. Sending a message: extracellular vesicles of pathogenic protozoan parasites. Nat Rev Microbiol. 2016;14(11):669–75. Epub 2016/09/13. doi: 10.1038/nrmicro.2016.110 . 27615028

[27] Liebana-Jordan M, Brotons B, Falcon-Perez JM, Gonzalez E. Extracellular Vesicles in the Fungi Kingdom. Int J Mol Sci. 2021;22(13). Epub 2021/07/21. doi: 10.3390/ijms22137221 ; PubMed Central PMCID: PMC8269022. 34281276 PMC8269022

[28] Cruz Camacho A, Alfandari D, Kozela E, Regev-Rudzki N. Biogenesis of extracellular vesicles in protozoan parasites: The ESCRT complex in the trafficking fast lane? PLoS Pathog. 2023;19(2):e1011140. Epub 2023/02/24. doi: 10.1371/journal.ppat.1011140 ; PubMed Central PMCID: PMC9949670. 36821560 PMC9949670

[29] Kim JH, Lee J, Park J, Gho YS. Gram-negative and Gram-positive bacterial extracellular vesicles. Semin Cell Dev Biol. 2015;40:97–104. Epub 2015/02/24. doi: 10.1016/j.semcdb.2015.02.006 . 25704309

[30] Abellon-Ruiz J, Kaptan SS, Basle A, Claudi B, Bumann D, Kleinekathofer U, et al. Structural basis for maintenance of bacterial outer membrane lipid asymmetry. Nat Microbiol. 2017;2(12):1616–23. Epub 2017/10/19. doi: 10.1038/s41564-017-0046-x . 29038444

[31] May KL, Grabowicz M. The bacterial outer membrane is an evolving antibiotic barrier. Proc Natl Acad Sci U S A. 2018;115(36):8852–4. Epub 2018/08/25. doi: 10.1073/pnas.1812779115 ; PubMed Central PMCID: PMC6130387. 30139916 PMC6130387

[32] Miller WR, Bayer AS, Arias CA. Mechanism of Action and Resistance to Daptomycin in Staphylococcus aureus and Enterococci. Cold Spring Harb Perspect Med. 2016;6(11). Epub 2016/11/03. doi: 10.1101/cshperspect.a026997 ; PubMed Central PMCID: PMC5088507. 27580748 PMC5088507

[33] Trimble MJ, Mlynarcik P, Kolar M, Hancock RE. Polymyxin: Alternative Mechanisms of Action and Resistance. Cold Spring Harb Perspect Med. 2016;6(10). Epub 2016/08/10. doi: 10.1101/cshperspect.a025288 ; PubMed Central PMCID: PMC5046685. 27503996 PMC5046685

[34] Manning AJ, Kuehn MJ. Contribution of bacterial outer membrane vesicles to innate bacterial defense. BMC Microbiol. 2011;11:258. Epub 2011/12/03. doi: 10.1186/1471-2180-11-258 ; PubMed Central PMCID: PMC3248377. 22133164 PMC3248377

[35] Kulkarni HM, Nagaraj R, Jagannadham MV. Protective role of E. coli outer membrane vesicles against antibiotics. Microbiol Res. 2015;181:1–7. Epub 2015/12/08. doi: 10.1016/j.micres.2015.07.008 . 26640046

[36] Kulkarni HM, Swamy Ch V, Jagannadham MV. Molecular characterization and functional analysis of outer membrane vesicles from the antarctic bacterium Pseudomonas syringae suggest a possible response to environmental conditions. J Proteome Res. 2014;13(3):1345–58. Epub 2014/01/21. doi: 10.1021/pr4009223 . 24437924

[37] Kadurugamuwa JL, Beveridge TJ. Bacteriolytic effect of membrane vesicles from Pseudomonas aeruginosa on other bacteria including pathogens: conceptually new antibiotics. J Bacteriol. 1996;178(10):2767–74. Epub 1996/05/01. doi: 10.1128/jb.178.10.2767-2774.1996 ; PubMed Central PMCID: PMC178010. 8631663 PMC178010

[38] Kadurugamuwa JL, Beveridge TJ. Delivery of the non-membrane-permeative antibiotic gentamicin into mammalian cells by using Shigella flexneri membrane vesicles. Antimicrob Agents Chemother. 1998;42(6):1476–83. Epub 1998/06/13. doi: 10.1128/AAC.42.6.1476 ; PubMed Central PMCID: PMC105625. 9624497 PMC105625

[39] Huang W, Zhang Q, Li W, Yuan M, Zhou J, Hua L, et al. Development of novel nanoantibiotics using an outer membrane vesicle-based drug efflux mechanism. J Control Release. 2020;317:1–22. Epub 2019/11/19. doi: 10.1016/j.jconrel.2019.11.017 . 31738965

[40] Medvedeva ES, Baranova NB, Mouzykantov AA, Grigorieva TY, Davydova MN, Trushin MV, et al. Adaptation of mycoplasmas to antimicrobial agents: Acholeplasma laidlawii extracellular vesicles mediate the export of ciprofloxacin and a mutant gene related to the antibiotic target. Sci World J. 2014;2014:150615. Epub 2014/03/08. doi: 10.1155/2014/150615 ; PubMed Central PMCID: PMC3925563. 24605048 PMC3925563

[41] Devos S, Van Putte W, Vitse J, Van Driessche G, Stremersch S, Van Den Broek W, et al. Membrane vesicle secretion and prophage induction in multidrug-resistant Stenotrophomonas maltophilia in response to ciprofloxacin stress. Environ Microbiol. 2017;19(10):3930–7. Epub 2017/05/11. doi: 10.1111/1462-2920.13793 . 28488744

[42] Dhital S, Deo P, Bharathwaj M, Horan K, Nickson J, Azad M, et al. Neisseria gonorrhoeae-derived outer membrane vesicles package beta-lactamases to promote antibiotic resistance. Microlife. 2022;3:uqac013. Epub 2023/05/24. doi: 10.1093/femsml/uqac013 ; PubMed Central PMCID: PMC10117772. 37223348 PMC10117772

[43] Ciofu O, Beveridge TJ, Kadurugamuwa J, Walther-Rasmussen J, Hoiby N. Chromosomal beta-lactamase is packaged into membrane vesicles and secreted from Pseudomonas aeruginosa. The Journal of Antimicrobial Chemotherapy 2000;45(1):9–13. Epub 2000/01/11. doi: 10.1093/jac/45.1.9 . 10629007

[44] Chattopadhyay MK, Jaganandham MV. Vesicles-mediated resistance to antibiotics in bacteria. Front Microbiol. 2015;6:758. Epub 2015/08/11. doi: 10.3389/fmicb.2015.00758 ; PubMed Central PMCID: PMC4511839. 26257725 PMC4511839

[45] Liao YT, Kuo SC, Chiang MH, Lee YT, Sung WC, Chen YH, et al. Acinetobacter baumannii Extracellular OXA-58 Is Primarily and Selectively Released via Outer Membrane Vesicles after Sec-Dependent Periplasmic Translocation. Antimicrob Agents Chemother. 2015;59(12):7346–54. Epub 2015/09/16. doi: 10.1128/AAC.01343-15 ; PubMed Central PMCID: PMC4649246. 26369971 PMC4649246

[46] Lopez C, Prunotto A, Bahr G, Bonomo RA, Gonzalez LJ, Dal Peraro M, et al. Specific Protein-Membrane Interactions Promote Packaging of Metallo-beta-Lactamases into Outer Membrane Vesicles. Antimicrob Agents Chemother. 2021;65(10):e0050721. Epub 2021/07/27. doi: 10.1128/AAC.00507-21 ; PubMed Central PMCID: PMC8448117. 34310214 PMC8448117

[47] Schaar V, Nordstrom T, Morgelin M, Riesbeck K. Moraxella catarrhalis outer membrane vesicles carry beta-lactamase and promote survival of Streptococcus pneumoniae and Haemophilus influenzae by inactivating amoxicillin. Antimicrob Agents Chemother. 2011;55(8):3845–53. Epub 2011/05/18. doi: 10.1128/AAC.01772-10 ; PubMed Central PMCID: PMC3147650. 21576428 PMC3147650

[48] Schaar V, Paulsson M, Morgelin M, Riesbeck K. Outer membrane vesicles shield Moraxella catarrhalis beta-lactamase from neutralization by serum IgG. J Antimicrob Chemother. 2013;68(3):593–600. Epub 2012/11/28. doi: 10.1093/jac/dks444 . 23184710

[49] Schaar V, Uddback I, Nordstrom T, Riesbeck K. Group A streptococci are protected from amoxicillin-mediated killing by vesicles containing beta-lactamase derived from Haemophilus influenzae. J Antimicrob Chemother. 2014;69(1):117–20. Epub 2013/08/06. doi: 10.1093/jac/dkt307 . 23912886

[50] Stentz R, Horn N, Cross K, Salt L, Brearley C, Livermore DM, et al. Cephalosporinases associated with outer membrane vesicles released by Bacteroides spp. protect gut pathogens and commensals against beta-lactam antibiotics. J Antimicrob Chemother. 2015;70(3):701–9. Epub 2014/11/30. doi: 10.1093/jac/dku466 ; PubMed Central PMCID: PMC4319488. 25433011 PMC4319488

[51] Lee AR, Park SB, Kim SW, Jung JW, Chun JH, Kim J, et al. Membrane vesicles from antibiotic-resistant Staphylococcus aureus transfer antibiotic-resistance to antibiotic-susceptible Escherichia coli. Journal of Applied Microbiology 2022;132(4):2746–59. Epub 2022/01/13. doi: 10.1111/jam.15449 ; PubMed Central PMCID: PMC9306644. 35019198 PMC9306644

[52] Vergalli J, Bodrenko IV, Masi M, Moynie L, Acosta-Gutierrez S, Naismith JH, et al. Porins and small-molecule translocation across the outer membrane of Gram-negative bacteria. Nat Rev Microbiol. 2020;18(3):164–76. Epub 2019/12/04. doi: 10.1038/s41579-019-0294-2 . 31792365

[53] Kim SW, Lee JS, Park SB, Lee AR, Jung JW, Chun JH, et al. The Importance of Porins and beta-Lactamase in Outer Membrane Vesicles on the Hydrolysis of beta-Lactam Antibiotics. Int J Mol Sci. 2020;21(8). Epub 2020/04/23. doi: 10.3390/ijms21082822 ; PubMed Central PMCID: PMC7215730. 32316670 PMC7215730

[54] Zhang X, Qian C, Tang M, Zeng W, Kong J, Fu C, et al. Carbapenemase-loaded outer membrane vesicles protect Pseudomonas aeruginosa by degrading imipenem and promoting mutation of antimicrobial resistance gene. Drug Resistance Updates 2023;68:100952. Epub 2023/02/23. doi: 10.1016/j.drup.2023.100952 . 36812748

[55] Chen LJ, Jing XP, Meng DL, Wu TT, Zhou H, Sun RL, et al. Newly Detected Transmission of bla(KPC-2) by Outer Membrane Vesicles in Klebsiella Pneumoniae. Current Medical Science 2023;43(1):80–5. Epub 2023/01/06. doi: 10.1007/s11596-022-2680-7 . 36602673

[56] Tang B, Yang A, Liu P, Wang Z, Jian Z, Chen X, et al. Outer Membrane Vesicles Transmitting bla(NDM-1) Mediate the Emergence of Carbapenem-Resistant Hypervirulent Klebsiella pneumoniae. Antimicrob Agents Chemother. 2023;67(5):e0144422. Epub 2023/04/14. doi: 10.1128/aac.01444-22 ; PubMed Central PMCID: PMC10190253. 37052502 PMC10190253

[57] Rumbo C, Fernandez-Moreira E, Merino M, Poza M, Mendez JA, Soares NC, et al. Horizontal transfer of the OXA-24 carbapenemase gene via outer membrane vesicles: a new mechanism of dissemination of carbapenem resistance genes in Acinetobacter baumannii. Antimicrob Agents Chemother. 2011;55(7):3084–90. Epub 2011/04/27. doi: 10.1128/AAC.00929-10 ; PubMed Central PMCID: PMC3122458. 21518847 PMC3122458

[58] Chatterjee S, Mondal A, Mitra S, Basu S. Acinetobacter baumannii transfers the blaNDM-1 gene via outer membrane vesicles. J Antimicrob Chemother. 2017;72(8):2201–7. Epub 2017/05/16. doi: 10.1093/jac/dkx131 . 28505330

[59] Li C, Wen R, Mu R, Chen X, Ma P, Gu K, et al. Outer Membrane Vesicles of Avian PathogenicEscherichia coli Mediate the Horizontal Transmission of bla(CTX-M-55). Pathogens. 2022;11(4). Epub 2022/04/24. doi: 10.3390/pathogens11040481 ; PubMed Central PMCID: PMC9025603. 35456156 PMC9025603

[60] Bielaszewska M, Daniel O, Karch H, Mellmann A. Dissemination of the blaCTX-M-15 gene among Enterobacteriaceae via outer membrane vesicles. J Antimicrob Chemother. 2020;75(9):2442–51. Epub 2020/06/21. doi: 10.1093/jac/dkaa214 . 32562546

[61] Fulsundar S, Harms K, Flaten GE, Johnsen PJ, Chopade BA, Nielsen KM. Gene transfer potential of outer membrane vesicles of Acinetobacter baylyi and effects of stress on vesiculation. Appl Environ Microbiol. 2014;80(11):3469–83. Epub 2014/03/25. doi: 10.1128/AEM.04248-13 ; PubMed Central PMCID: PMC4018862. 24657872 PMC4018862

[62] Xu J, Mei C, Zhi Y, Liang ZX, Zhang X, Wang HJ. Comparative Genomics Analysis and Outer Membrane Vesicle-Mediated Horizontal Antibiotic-Resistance Gene Transfer in Avibacterium paragallinarum. Microbiol Spectr. 2022;10(5):e0137922. Epub 2022/08/25. doi: 10.1128/spectrum.01379-22 ; PubMed Central PMCID: PMC9603892. 36000914 PMC9603892

[63] Tran F, Boedicker JQ. Plasmid Characteristics Modulate the Propensity of Gene Exchange in Bacterial Vesicles. J Bacteriol. 2019;201(7). Epub 2019/01/24. doi: 10.1128/JB.00430-18 ; PubMed Central PMCID: PMC6416910. 30670543 PMC6416910

[64] Johnston EL, Zavan L, Bitto NJ, Petrovski S, Hill AF, Kaparakis-Liaskos M. Planktonic and Biofilm-Derived Pseudomonas aeruginosa Outer Membrane Vesicles Facilitate Horizontal Gene Transfer of Plasmid DNA. Microbiol Spectr. 2023;11(2):e0517922. Epub 2023/03/23. doi: 10.1128/spectrum.05179-22 ; PubMed Central PMCID: PMC10100964. 36946779 PMC10100964

[65] Kulig K, Karnas E, Woznicka O, Kuleta P, Zuba-Surma E, Pyza E, et al. Insight Into the Properties and Immunoregulatory Effect of Extracellular Vesicles Produced by Candida glabrata, Candida parapsilosis, and Candida tropicalis Biofilms. Front Cell Infect Microbiol. 2022;12:879237. Epub 2022/06/24. doi: 10.3389/fcimb.2022.879237 ; PubMed Central PMCID: PMC9207348. 35734578 PMC9207348

[66] Zarnowski R, Sanchez H, Covelli AS, Dominguez E, Jaromin A, Bernhardt J, et al. Candida albicans biofilm-induced vesicles confer drug resistance through matrix biogenesis. PLoS Biol. 2018;16(10):e2006872. Epub 2018/10/09. doi: 10.1371/journal.pbio.2006872 ; PubMed Central PMCID: PMC6209495. 30296253 PMC6209495

[67] Zarnowski R, Noll A, Chevrette MG, Sanchez H, Jones R, Anhalt H, et al. Coordination of fungal biofilm development by extracellular vesicle cargo. Nat Commun. 2021;12(1):6235. Epub 2021/10/31. doi: 10.1038/s41467-021-26525-z ; PubMed Central PMCID: PMC8556236. 34716343 PMC8556236

[68] Zarnowski R, Sanchez H, Jaromin A, Zarnowska UJ, Nett JE, Mitchell AP, et al. A common vesicle proteome drives fungal biofilm development. Proc Natl Acad Sci U S A. 2022;119(38):e2211424119. Epub 2022/09/13. doi: 10.1073/pnas.2211424119 ; PubMed Central PMCID: PMC9501958. 36095193 PMC9501958

[69] Zhao M, Zhang F, Zarnowski R, Barns K, Jones R, Fossen J, et al. Turbinmicin inhibits Candida biofilm growth by disrupting fungal vesicle-mediated trafficking. J Clin Invest. 2021;131(5). Epub 2020/12/30. doi: 10.1172/JCI145123 ; PubMed Central PMCID: PMC7919718. 33373326 PMC7919718

[70] Amatuzzi RF, Zamith-Miranda D, Munhoz da Rocha IF, Lucena ACR, de Toledo Martins S, Streit R, et al. Caspofungin Affects Extracellular Vesicle Production and Cargo in Candida auris. J Fungi (Basel). 2022;8(10). Epub 2022/10/28. doi: 10.3390/jof8100990 ; PubMed Central PMCID: PMC9605528. 36294557 PMC9605528

[71] Zhao K, Bleackley M, Chisanga D, Gangoda L, Fonseka P, Liem M, et al. Extracellular vesicles secreted by Saccharomyces cerevisiae are involved in cell wall remodelling. Commun Biol. 2019;2:305. Epub 2019/08/21. doi: 10.1038/s42003-019-0538-8 ; PubMed Central PMCID: PMC6688994. 31428693 PMC6688994

[72] Martinez-Lopez R, Hernaez ML, Redondo E, Calvo G, Radau S, Pardo M, et al. Candida albicans Hyphal Extracellular Vesicles Are Different from Yeast Ones, Carrying an Active Proteasome Complex and Showing a Different Role in Host Immune Response. Microbiol Spectr. 2022;10(3):e0069822. Epub 2022/05/24. doi: 10.1128/spectrum.00698-22 ; PubMed Central PMCID: PMC9241596. 35604172 PMC9241596

[73] Chan W, Chow FW, Tsang CC, Liu X, Yao W, Chan TT, et al. Induction of amphotericin B resistance in susceptible Candida auris by extracellular vesicles. Emerg Microbes Infect. 2022;11(1):1900–9. Epub 2022/07/06. doi: 10.1080/22221751.2022.2098058 ; PubMed Central PMCID: PMC9341352. 35786393 PMC9341352

[74] Whaley SG, Berkow EL, Rybak JM, Nishimoto AT, Barker KS, Rogers PD. Azole Antifungal Resistance in Candida albicans and Emerging Non-albicans Candida Species. Front Microbiol. 2016;7:2173. Epub 2017/01/28. doi: 10.3389/fmicb.2016.02173 ; PubMed Central PMCID: PMC5226953. 28127295 PMC5226953

[75] Verweij PE, Snelders E, Kema GH, Mellado E, Melchers WJ. Azole resistance in Aspergillus fumigatus: a side-effect of environmental fungicide use? Lancet Infect Dis. 2009;9(12):789–95. Epub 2009/11/21. doi: 10.1016/S1473-3099(09)70265-8 . 19926038

[76] Cheong JW, McCormack J. Fluconazole resistance in cryptococcal disease: emerging or intrinsic? Med Mycol. 2013;51(3):261–9. Epub 2012/09/20. doi: 10.3109/13693786.2012.715763 . 22989195

[77] Revie NM, Iyer KR, Robbins N, Cowen LE. Antifungal drug resistance: evolution, mechanisms and impact. Curr Opin Microbiol. 2018;45:70–6. Epub 2018/03/17. doi: 10.1016/j.mib.2018.02.005 ; PubMed Central PMCID: PMC6135714. 29547801 PMC6135714

[78] Berman J, Krysan DJ. Drug resistance and tolerance in fungi. Nat Rev Microbiol. 2020;18(6):319–31. Epub 2020/02/13. doi: 10.1038/s41579-019-0322-2 ; PubMed Central PMCID: PMC7231573. 32047294 PMC7231573

[79] Rizzo J, Trottier A, Moyrand F, Coppee JY, Maufrais C, Zimbres ACG, et al. Coregulation of extracellular vesicle production and fluconazole susceptibility in Cryptococcus neoformans. MBio. 2023;14(4):e0087023. Epub 2023/06/13. doi: 10.1128/mbio.00870-23 ; PubMed Central PMCID: PMC10470540. 37310732 PMC10470540

[80] Marcilla A, Martin-Jaular L, Trelis M, de Menezes-Neto A, Osuna A, Bernal D, et al. Extracellular vesicles in parasitic diseases. J Extracell Vesicles. 2014;3:25040. Epub 2014/12/30. doi: 10.3402/jev.v3.25040 ; PubMed Central PMCID: PMC4275648. 25536932 PMC4275648

[81] Marti M, Johnson PJ. Emerging roles for extracellular vesicles in parasitic infections. Curr Opin Microbiol. 2016;32:66–70. Epub 2016/05/22. doi: 10.1016/j.mib.2016.04.008 ; PubMed Central PMCID: PMC6445373. 27208506 PMC6445373

[82] Gazzinelli-Guimaraes PH, Nutman TB. Helminth parasites and immune regulation. F1000Res. 2018;7. Epub 2018/11/13. doi: 10.12688/f1000research.15596.1 ; PubMed Central PMCID: PMC6206608. 30416709 PMC6206608

[83] Regev-Rudzki N, Wilson DW, Carvalho TG, Sisquella X, Coleman BM, Rug M, et al. Cell-cell communication between malaria-infected red blood cells via exosome-like vesicles. Cell. 2013;153(5):1120–33. Epub 2013/05/21. doi: 10.1016/j.cell.2013.04.029 . 23683579

[84] Douanne N, Dong G, Amin A, Bernardo L, Blanchette M, Langlais D, et al. Leishmania parasites exchange drug-resistance genes through extracellular vesicles. Cell Rep. 2022;40(3):111121. Epub 2022/07/21. doi: 10.1016/j.celrep.2022.111121 . 35858561

[85] Szempruch AJ, Sykes SE, Kieft R, Dennison L, Becker AC, Gartrell A, et al. Extracellular Vesicles from Trypanosoma brucei Mediate Virulence Factor Transfer and Cause Host Anemia. Cell. 2016;164(1–2):246–57. Epub 2016/01/16. doi: 10.1016/j.cell.2015.11.051 ; PubMed Central PMCID: PMC4715261. 26771494 PMC4715261

[86] Douanne N, Dong G, Douanne M, Olivier M, Fernandez-Prada C. Unravelling the proteomic signature of extracellular vesicles released by drug-resistant Leishmania infantum parasites. PLoS Negl Trop Dis. 2020;14(7):e0008439. Epub 2020/07/07. doi: 10.1371/journal.pntd.0008439 ; PubMed Central PMCID: PMC7365475. 32628683 PMC7365475

[87] Perez J, Contreras-Moreno FJ, Marcos-Torres FJ, Moraleda-Munoz A, Munoz-Dorado J. The antibiotic crisis: How bacterial predators can help. Comput Struct Biotechnol J. 2020;18:2547–55. Epub 2020/10/10. doi: 10.1016/j.csbj.2020.09.010 ; PubMed Central PMCID: PMC7522538. 33033577 PMC7522538

[88] Chen YC, Kalawong R, Toyofuku M, Eberl L. The role of peptidoglycan hydrolases in the formation and toxicity of Pseudomonas aeruginosa membrane vesicles. Microlife. 2022;3:uqac009. Epub 2023/05/25. doi: 10.1093/femsml/uqac009 ; PubMed Central PMCID: PMC10117874. 37229443 PMC10117874

[89] Wang Y, Hoffmann JP, Chou CW, Honer Zu Bentrup K, Fuselier JA, Bitoun JP, et al. Burkholderia thailandensis outer membrane vesicles exert antimicrobial activity against drug-resistant and competitor microbial species. J Microbiol. 2020;58(7):550–62. Epub 2020/04/14. doi: 10.1007/s12275-020-0028-1 . 32281050

[90] Evans AGL, Davey HM, Cookson A, Currinn H, Cooke-Fox G, Stanczyk PJ, et al. Predatory activity of Myxococcus xanthus outer-membrane vesicles and properties of their hydrolase cargo. Microbiology (Reading). 2012;158(Pt 11):2742–52. Epub 2012/09/15. doi: 10.1099/mic.0.060343-0 . 22977088

[91] Lehar SM, Pillow T, Xu M, Staben L, Kajihara KK, Vandlen R, et al. Novel antibody-antibiotic conjugate eliminates intracellular S. aureus. Nature. 2015;527(7578):323–8. Epub 2015/11/05. doi: 10.1038/nature16057 . 26536114

[92] Goes A, Lapuhs P, Kuhn T, Schulz E, Richter R, Panter F, et al. Myxobacteria-Derived Outer Membrane Vesicles: Potential Applicability Against Intracellular Infections. Cells. 2020;9(1). Epub 2020/01/17. doi: 10.3390/cells9010194 ; PubMed Central PMCID: PMC7017139. 31940898 PMC7017139

[93] Meers PR, Liu C, Chen R, Bartos W, Davis J, Dziedzic N, et al. Vesicular Delivery of the Antifungal Antibiotics of Lysobacter enzymogenes C3. Appl Environ Microbiol. 2018;84(20). Epub 2018/08/12. doi: 10.1128/AEM.01353-18 ; PubMed Central PMCID: PMC6182896. 30097441 PMC6182896

[94] Yue H, Jiang J, Taylor AJ, Leite AL, Dodds ED, Du L. Outer Membrane Vesicle-Mediated Codelivery of the Antifungal HSAF Metabolites and Lytic Polysaccharide Monooxygenase in the Predatory Lysobacter enzymogenes. ACS Chem Biol. 2021;16(6):1079–89. Epub 2021/05/26. doi: 10.1021/acschembio.1c00260 ; PubMed Central PMCID: PMC8797504. 34032403 PMC8797504

[95] Collins SM, Brown AC. Bacterial Outer Membrane Vesicles as Antibiotic Delivery Vehicles. Front Immunol. 2021;12:733064. Epub 2021/10/08. doi: 10.3389/fimmu.2021.733064 ; PubMed Central PMCID: PMC8488215. 34616401 PMC8488215

[96] Alves NJ, Turner KB, Daniele MA, Oh E, Medintz IL, Walper SA. Bacterial Nanobioreactors—Directing Enzyme Packaging into Bacterial Outer Membrane Vesicles. ACS Appl Mater Interfaces. 2015;7(44):24963–72. Epub 2015/10/20. doi: 10.1021/acsami.5b08811 . 26479678

[97] Bomberger JM, Maceachran DP, Coutermarsh BA, Ye S, O’Toole GA, Stanton BA. Long-distance delivery of bacterial virulence factors by Pseudomonas aeruginosa outer membrane vesicles. PLoS Pathog. 2009;5(4):e1000382. Epub 2009/04/11. doi: 10.1371/journal.ppat.1000382 ; PubMed Central PMCID: PMC2661024. 19360133 PMC2661024

[98] Tashiro Y, Hasegawa Y, Shintani M, Takaki K, Ohkuma M, Kimbara K, et al. Interaction of Bacterial Membrane Vesicles with Specific Species and Their Potential for Delivery to Target Cells. Front Microbiol. 2017;8:571. Epub 2017/04/26. doi: 10.3389/fmicb.2017.00571 ; PubMed Central PMCID: PMC5383704. 28439261 PMC5383704

[99] Nikaido H. Molecular basis of bacterial outer membrane permeability revisited. Microbiol Mol Biol Rev. 2003;67(4):593–656. Epub 2003/12/11. doi: 10.1128/MMBR.67.4.593-656.2003 ; PubMed Central PMCID: PMC309051. 14665678 PMC309051

[100] Delcour AH. Outer membrane permeability and antibiotic resistance. Biochim Biophys Acta. 2009;1794(5):808–16. Epub 2008/12/23. doi: 10.1016/j.bbapap.2008.11.005 ; PubMed Central PMCID: PMC2696358. 19100346 PMC2696358

[101] Richter R, Kamal MAM, Koch M, Niebuur BJ, Huber AL, Goes A, et al. An Outer Membrane Vesicle-Based Permeation Assay (OMPA) for Assessing Bacterial Bioavailability. Adv Healthc Mater. 2022;11(5):e2101180. Epub 2021/10/07. doi: 10.1002/adhm.202101180 . 34614289 PMC11468809

[102] Ferreira RJ, Kasson PM. Antibiotic Uptake Across Gram-Negative Outer Membranes: Better Predictions Towards Better Antibiotics. ACS Infect Dis. 2019;5(12):2096–104. Epub 2019/10/09. doi: 10.1021/acsinfecdis.9b00201 . 31593635

